# Extensive Polycistronism and Antisense Transcription in the Mammalian *Hox* Clusters

**DOI:** 10.1371/journal.pone.0000356

**Published:** 2007-04-04

**Authors:** Gaëll Mainguy, Jan Koster, Joost Woltering, Hans Jansen, Antony Durston

**Affiliations:** 1 Hubrecht Laboratory, Center for Biomedical Genetics, Utrecht, The Netherlands; 2 Department of Human Genetics, Academic Medical Center, University of Amsterdam, Amsterdam, The Netherlands; Baylor College of Medicine, United States of America

## Abstract

The *Hox* clusters play a crucial role in body patterning during animal development. They encode both *Hox* transcription factor and micro-RNA genes that are activated in a precise temporal and spatial sequence that follows their chromosomal order. These remarkable collinear properties confer functional unit status for *Hox* clusters. We developed the TranscriptView platform to establish high resolution transcriptional profiling and report here that transcription in the *Hox* clusters is far more complex than previously described in both human and mouse. Unannotated transcripts can represent up to 60% of the total transcriptional output of a cluster. In particular, we identified 14 non-coding Transcriptional Units antisense to *Hox* genes, 10 of which (70%) have a detectable mouse homolog. Most of these Transcriptional Units in both human and mouse present conserved sizeable sequences (>40 bp) overlapping *Hox* transcripts, suggesting that these *Hox* antisense transcripts are functional. *Hox* clusters also display at least seven polycistronic clusters, i.e., different genes being co-transcribed on long isoforms (up to 30 kb). This work provides a reevaluated framework for understanding *Hox* gene function and dys-function. Such extensive transcriptions may provide a structural explanation for *Hox* clustering.

## Introduction

Hox clusters are amongst the most remarkable genomic objects, the structure and function of which are crucial to understand, as Hox clusters are implicated in a growing number of diseases from cancers to congenital malformations [1]. Mammals possess four similar Hox clusters, HoxA, HoxB, HoxC and HoxD, located on different chromosomes, consisting of 9 to 11 *Hox* genes arranged in tandem. The order of *Hox* genes along the chromosome corresponds to the order in which they act along the body axes and this collinear property links clustering to function emphasizing that Hox clusters are functional units [2]. The Hox clusters also contain 5 micro RNA (miRNA) genes intercalated at two homologous positions [3], [4]. The organization of Hox complexes is highly conserved in vertebrates and *Hox* and *mir* genes not only stay clustered but also in close proximity to each other despite their very complex and dynamic expression patterns, a property in apparent contradiction with the observation that the more complex the expression pattern of a gene is, the larger its flanking non coding DNA [5].

This apparent paradox raises the question of the selective pressure(s) at work for maintaining *Hox* and *mir* genes clustered. Current models propose that clustering is maintained *via* the sharing of *cis*-regulatory elements that control several *Hox* genes either locally or globally [6], [7], [2]. Other aspects of transcriptional structure could also be important. First, a case of polycistronism has been reported where *Hoxc6*, *Hoxc5* and *Hoxc4* are co-transcribed and gene-specific transcripts result from alternative splicing [8]. Notably, polycistronic Hox transcripts have also been reported in a number of crustaceans [9], indicating their importance in diverse metazoa. Second, a *Hoxa11* antisense RNA is transcribed immediately 5′ to *HoxA11* and is involved in its regulation [10]. Thus, Hox clusters present unusual transcriptional characteristics that may play an important role for Hox gene expression.

The transcriptional complexity of mammalian genomes is increasingly recognized [11] and data mining provides a suitable way to establish transcriptional structure of poorly expressed genes.Here we present a thorough analysis of the best described vertebrate (human and murine) Hox clusters.

## Results and Discussion

### The majority of the transcriptional activity of the Hox Clusters is not annotated

**Figure 1:**
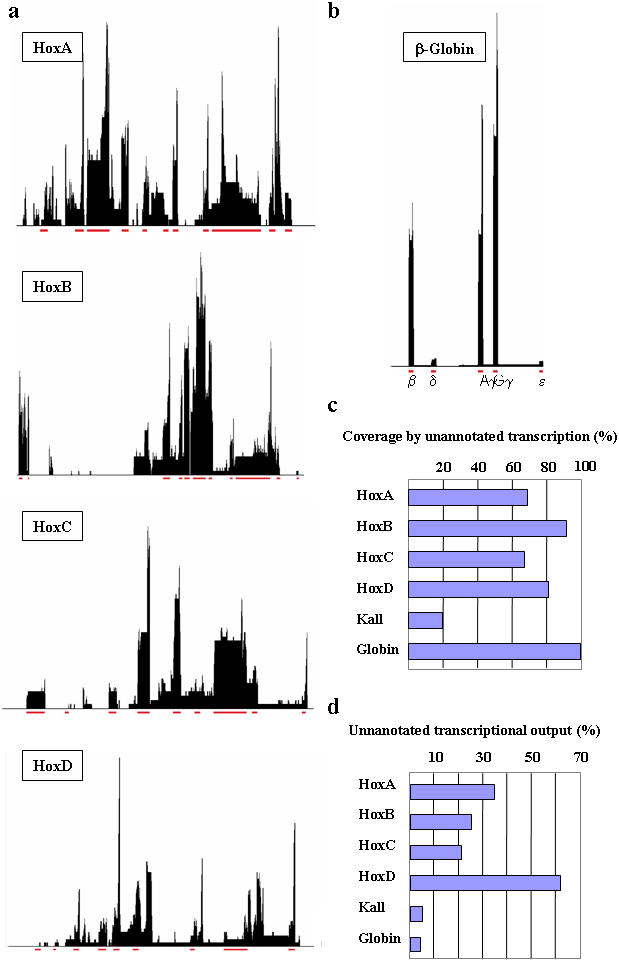
Transcription profiles of the Human Hox clusters. (a) Human *Hox* Cluster transcriptographs. (b) Transcriptograph of the human β-globin cluster. Note that despite an extensive transcription, the vast majority of sequences correspond to annotated genes. In 1a and 1b, annotations of genes in Refseq are depicted in red. (c) Proportion of the clusters that are primarily transcribed (d) Amount of transcription not currently annotated.

As the gene is a misleading concept we follow the unambiguous definitions proposed by the FANTOM consortium: A transcriptional unit (TU) is a segment of the genome flanked by the most distal exons from which transcripts are generated [12]. The transcripts sharing any exon are merged into a single TU. If two transcripts do not share any single exon, they constitute two different TUs, even if they overlap or if one is localized in the intron of the other. In particular, two transcripts on opposite strands always constitute two different TUs. Aligning the genome with all of the ESTs and mRNA provides a reliable method to delineate exons and deduce TU structures in the entire organism, independently of time and space and throughout its life cycle [13]. We computationally mined public mouse and human databases using a dedicated software platform, TranscriptView (see material and methods) and found that Hox cluster profiles are far more complex than annotated (fig 1a) but nonetheless very similar between human and mouse (see supporting online material). The importance of transcription beyond annotation has been established and 12.2% of the unannotated human chromosome 22 is transcribed [14]. In the Hox clusters, we found that this proportion ranged from 67% (HoxC) to 92% (HoxD) (fig 1c). Moreover, these unannotated transcripts can represent up to 60% of the total transcriptional output of a cluster (fig 1d), while it is a marginal phenomenon in two other clustered gene families, Globin and Kallikrein (<5%) (fig 1b,d). Kallikrein genes present a loosely clustered organization with 15 and 25 genes in human and mouse respectively, the function if any of the clustering being not known [15]. On the other hand, the β-Globin cluster is another example of functional clustering since β-Globin gene expression displays temporal collinearity. Even for the β-Globin cluster that presents extensive intergenic transcription ([16] and figure 1b,c), more than 95% of the transcribed sequences match annotated genes (see fig 1b,d). In general, the distribution of ESTs to genes is highly skewed as a large number of genes are represented by only one or a few transcripts [17], our results are therefore likely to be an underestimation.

**Figure 2:**
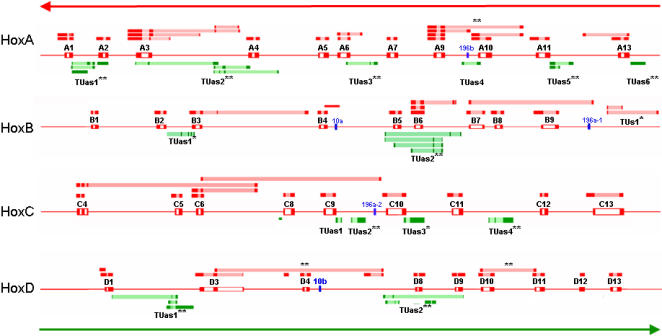
Synopsis of transcriptional activity in the Human Hox Clusters. Sense and antisense transcriptions are in red and green respectively. Dark and light shaded boxes represent exon and intron. Mir genes are in blue. The three long transcripts presenting a murine homolog with a conserved exon-exon boundary are headed by **. The 13 TUs (12 antisense and one sense) located at similar position in human and mouse are denoted by an asterisk (*) while the 10 TUs showing conservation are depicted with

In an effort to re-annotate the Hox clusters, we used the following strategy to establish TUs and discriminate functional RNAs. First, we restricted our analysis to spliced transcripts as splicing is evidence against genomic contamination and splice site asymmetry allows transcript orientation. As most of these transcripts are non-coding (see below), protein conservation was a useless criterion. To categorize the TUs along a scale of degree of confidence, we therefore focused on the exon-intron structure and nucleotide sequence conservation. In our analysis, **Transcript existence** (1) is defined by presence of multiple spliced transcripts in human databases, **Sequence conservation** (2) is observed when transcripts from two different species share a conserved sequence and **Exon-Exon structure conservation** (3) characterizes transcripts from two different species displaying the same intron boundaries. Our findings are summarized in tables 1 and 2 and are depicted in figure 2. This delineation of TU in the Hox clusters reveals the occurrence of two major phenomena, polycistronism and antisense transcription.

**Table 1:** Polycistronism in the Human Hox cluster

			Genes			Conservation	
	N Sequence	Reference Hs	Hox	miRNA	Category	Species	Refs
HoxA	1	BE549099	A10-9	mir-196b	3	Hs, Cp	X13536
HoxD	1	BQ722165	D4-3	mir-10b	3	Hs, Mm	BY724257
HoxD	1	BM903736	D11-10		3	Hs, Mm	BU510536
HoxB	*		B4	mir-10a	1	Dr	
HoxB	6	BX116422	B7-8-9	mir-196a1	1	Hs	
HoxC	2	BE464190	C6-8-9	mir-196a2	1	Hs	
HoxC	7	NM_014620	C6-5-4		1	Hs	

* Not present in the databases, identified by RT-PCR.

Species: Cp: Guineapig, Dr : Zebrafish, Hs : Human, Mm : Mouse.

**Table 2:** Sense AntiSense overlaps

	*cis*-encoded Antisenses					*trans*-encoded Antisenses			
Hox	Species°	Number*		Longest (nt)	Conservation**	Species AS	AS	Hs	Mm
Conserved SAS									
A3	Hs,Mm	3	3	88	**72/82**	Hs	AS B3	66/74	
B3	Hs,Mm	1	1	78	**75/78**	Hs	AS A3		
B5	Hs,Mm	1	1	100	**90/98**				
SAS in Human									
A4	Hs	1		369	**136/160**				
A6	Hs	3		107	**89/98**	Hs	AS B5	68/75	
A7	Hs	2		392	**181/194**				
A9	Hs	2		279	111/114	Mm	AS B2		38/40
A10	Hs	2		369	**127/144**				
B6	Hs	1		168	**152/168**				
C10	Hs	1		44	**41/41**				
D1	Hs	1		145	**105/123**				
D9	Hs	1		87	68/75				
SAS in Mouse									
A1	Mm	2		92	**74/84**				
A11	Mm	1		607	**577/613**	Mm	AS C11	103/121	108/121
B2	Mm	2		135	**115/122**				
C11	Mm	1		129	**128/129**				
Putative SAS°°									
*A2*	*?*	*2*		***-***	*74/80*				
*C9*	*?*	*3*		*-*	*76/85*				
SAS in trans									
B1						Mm	AS A1	54/62	55/62
B4						Hs, Mm	AS B5	66/73	73/79
D11						Mm	AS C11	52/57	
D3						Hs, Mm	AS A3/AS B2	41/44	107/126

° Species where the SAS is observed

* Number of independent SAS per gene.

** Nucleotide identity. In bold when found in mRNA, in gDNA otherwise.

°° Antisense sequences from human that are matching Hox mRNA from mouse

### Polycistronic clusters

A polycistronic cluster designates two or more genes co-transcribed from a single promoter, sharing a non-coding exon, and whose products are generated by alternative splicing [18]. An operon is a particular case where the mRNA retains the different products after splicing. In Mammals, both operons and polycistronic clusters are scarcely documented [18]. One clear example nonetheless, is the case of the *Hoxc4*, *Hoxc5* and *Hoxc6* genes that can be co-transcribed from a common promoter [8]. We found 22 Hox transcripts for which introns seem to encompass other genes. In three cases we could identify a homolog in rodent that presented a conserved exon-exon boundary (>85% identity over at least 60 nucleotides, see supporting online material). In total, multiple alignment and identification of orthologs provided support for the existence of seven polycistronic clusters which concern 38% (15/39) of the *Hox* genes (table 1).

Remarkably, the five miRNAs are located within introns of atypical transcripts and are therefore co-transcribed with *Hox* genes (figure 2). Evidence for *Hoxb4* and *mir-10a* was missing in the databases and we confirmed their co-transcription by RT-PCR, providing hereby an explanation for the observation that these two genes have markedly similar expression patterns [19]. More generally, co-transcription of mir and *Hox* genes gives a seductive framework to interpret the stability of *Hox* and *mir* gene positions relative to each other. Our results also shed light on the importance of splicing regulation within the Hox clusters, a conclusion in accordance with the recent finding that the knock out of the gene encoding the spliceosomal protein Sf3b1 leads to deregulation of *Hox* gene expressions and severe skeletal transformations [20].

### Widespread antisense transcription

Our analysis also revealed the existence of 15 TUs distinct from the *Hox* and *mir* genes that are poly-adenylated and alternatively spliced like genuine products of RNA Polymerase II. Most of these TUs (14/15) are transcribed antisense (AS) to *Hox* genes (see fig 2), and AS transcription can represent up to 38% of the spliced transcripts (38.46% for HoxA, 33.11% for HoxB, 13.16% for HoxC and 34.84% for HoxD). Cis-encoded antisenses and bidirectional promoters are now known to be abundant in the human genome [11]. Whereas most of the previously identified vertebrate AS transcripts encode proteins [21], [22], we did not detect any conserved open reading frames suggesting that all of the Hox AS TUs are non-coding (see methods). However, 12 AS TUs can also be assigned to mouse Hox clusters at similar positions and 10 human AS TUs (71%) have a detectable homolog in the mouse transcriptome (figure 2 and table 3, Sm) and they are therefore likely to be functional.

**Table 3:** Conservation of TU AS between human and mouse

	antisense transcipts correspondance		
AS-TU	Seq Human	Seq Mouse	identity
**A AS1**	BC031342	AW456363	56/58
**A AS2**	AK092154	AK028207	391/430 (3)
**A AS3**	AK091933	AK012572	365/404 (3)
**A AS5**	BC025338	MMU20369	279/326 (3)
**A AS6**	AK093987	AK033508	102/125, 64/70 (1)
**B AS2**	BE676309	AK012587	217/256 (7)
**C AS2**	BC044251	BC034904	211/232 (2)
**C AS4**	AK123741	AK035706	212/251(9)
**D AS1**	BC030713	W45744	44/49
**D AS2**	BC009347	AK054396	67/74

To date, AS RNAs have been implicated in various aspects of eukaryotic gene expression as diverse as genomic imprinting, RNA interference, translational regulation, alternative splicing, or RNA editing [21], [22], [23]. AS transcripts frequently originate from the same locus as sense transcripts and are called *cis*-encoded antisenses. They are thought to exert a control on RNA sense expression by sense-antisense (SAS) pairing [21], [22]. We searched for potential SAS contacts (>40 bp) and found that nine AS TUs (65%) have sequences reverse-complementary to twelve *Hox* mRNAs (table 2). This proportion is rather high as, on a genomic scale, natural AS transcription has been evaluated to target from 2 to 8% of the human genes [21], [22]. Similarly, in mouse sequences eight *Hox* genes are subjected to *cis*-antisense interactions, three of which, *Hoxa3*, *Hoxb3* and *Hoxb5*, present the same SAS in both human and mouse (table 2). The conservation of SAS sequences between human and mouse strongly supports the hypothesis that these AS TUs are functional. Moreover, all of the SAS overlap sequences are remarkably conserved in the other species suggesting that *cis*-encoded antisenses could target as many as 22 *Hox* genes (table 2). Besides these interactions, *trans*-encoded AS RNAs have also been reported where the AS transcript originates from a different locus and displays only partial complementarity with the sense transcript [23]. We identified 6 and 5 potential *trans*-interactions in human and mouse respectively (SAS contact; >40 nucleotides, >85% identity) (table 2). These SAS interactions usually occur within a paralog group (A1/B1, A3/B3 or A11/C11/D11) but there are three noteworthy exceptions (B4/B5, B2/A9 and B2/D3). Remarkably, antisense transcripts with the potential to recognize *Hoxb4* and *Hoxd3* in *trans* are present in both human and mouse.

### Functional clustering and extensive transcription correlate with absence of transposons

**Figure 3:**
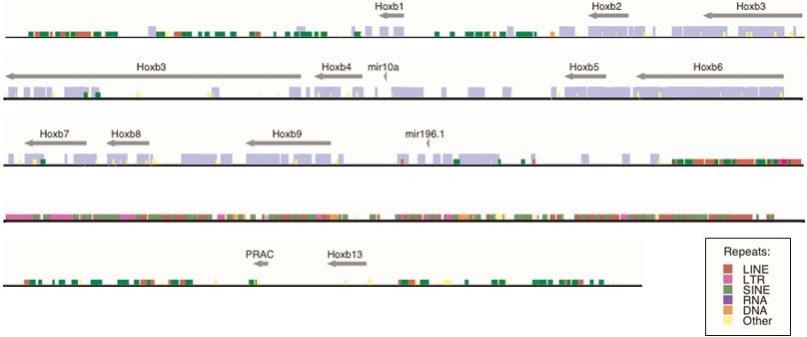
Transposons are excluded from transcribed regions. Human HoxB cluster has been repeat-masked. Deduced exons retrieved from TranscriptView are in blue. Human repeats are depicted by colored squares. Note that simple repeats, represented by yellow squares are distributed throughout the HoxB cluster independently of the gene content.

Our analysis suggests that, in addition to the sharing of *cis*-regulatory elements, the existence of operons, polycistronism and antisense-sense pairing provide additional constraints for maintaining Hox clusters as functional units. If this were the case, exogenous start and stop transcription signals would be highly counter-selected. Indeed, the four Hox clusters are by far the most repeat-poor regions of the genome in both human and mouse, and the current explanation is that insertions would interfere with the dense network of *cis*-interactions [24]. We analyzed the repeat distribution and found that transposons are virtually absent from transcribed regions but that they can accumulate within the clusters at untranscribed regions. The HoxB cluster provides a threefold example of this mutual exclusiveness between transposons and transcription (see figure 3, and see supporting online material for the other clusters). In both human and mouse, the intergenic region between *Hoxb1* and *Hoxb2* is notably not transcribed (see fig 1) and has been independently colonized by SINEs (13 in human, 17 in mouse). (2) The sequence upstream of *Hoxb9* is massively filled with repeats as *Hoxb13* is drifting away (Hs:107 SINEs, 31 LTRs, 61 LINEs; Mm: 113 SINEs, 18 LTRs, 14 LINEs). (3) But reciprocally, the posterior limit of repeat accumulation does not coincide with *Hoxb9* but with the non-coding TU that is upstream of it (figure 3). Moreover, whereas transposons are indeed very rare, on the other hand simple repeats of di- or tri-nucleotides are found throughout the Hox clusters (figure 3) arguing against the preeminence of sequence disruption *per se*. An alternative explanation could be that transposons are counter selected for their potential to interfere with transcription. Incidentally, this inverted correlation supports the hypothesis that these non-coding transcription products are functional.

### Concluding Remarks

Our analysis confers on Hox clusters the status of the most complex objects reported to date in mammals in terms of both polycistronism and antisense and suggests that, in addition to enhancer sharing, these mechanisms provide additional constraints for maintaining Hox clusters as functional units. There is increasing recognition that the production of RNA transcripts from both orientations can produce coordinate regulation and since mammalian mRNAs that form sense-antisense pairs frequently exhibit reciprocal expression patterns [21] it is tempting to speculate that antisense transcription in the Hox Clusters is instrumental in establishing limits of gene expression. In conclusion, by unraveling the complex transcriptional organisation of the Hox clusters, our analysis blurs the traditional view of *Hox* genes and provides a reevaluated framework for understanding *Hox* gene function and dys-function.

## Methods

### The TranscriptView software platform

We used the TranscriptView software platform to obtain and manipulate clusters of human expressed sequences aligned to genomic DNA. TranscriptView makes use of public genome alignment data for EST and mRNA sequences generated with BLAT by the UCSC genome consortium (http://genome.ucsc.edu/). The BLAT program is specifically designed for transcript to genome alignments making it possible to align large collections of sequences to the genome [25]. Expressed sequences are compared to the human genome to find high quality hits, and are then aligned to it using a spliced alignment model that allows long gaps, for modeling introns. The maximum intron length allowed by BLAT is 500,000 bases. When a single EST aligned in multiple places, the alignment having the highest base identity is identified. Low-quality sequence ends that disagree with the DNA are trimmed. Only alignments having a base identity level within 0.5% of the best and at least 96% base identity with the genomic sequence are kept (http://genome.ucsc.edu/cgi-bin/hgTrackUi?g = est). Further, expressed sequences aligning to two or more chromosomes are discarded as suspected chimeras. Overlapping expressed sequences and corresponding genomic sequences are multiply aligned. Positions on the genomic sequence in which there is at least one expressed sequence that opens or closes a long gap, are considered splice sites. The exact position of the splice sites is determined taking the GT...AG rule into consideration as described in [22]. The list of all of the alignment boundaries is generated allowing a quantitative determination of the transcriptional status of any genomic segment at the base pair level. The deduced Exon-intron organization and the orientation when available are also accessible through the TranscriptView graphical interface.

### Datasets

Using BLAT we retrieved 2630 Ests and mRNA sequences that aligned with the human Hox clusters (HoxA 837, HoxB 807, HoxC 441, HoxD 545). The distribution of this primary set is described in figure 1. As similarities of sequence within a cluster of tandemly repeated genes can be a source of misalignment we compared our results with two other clustered family of genes, Kallikrein and β-Globin clusters. Subsequent analysis of polycistronism and antisense was restricted to spliced sequences that account for ca. 25% of the primary set (HoxA 202, HoxB 241, HoxC 127, HoxD 133).

### TU annotations and transcript analysis

Among this secondary set, 96 sequences displayed at least one intron longer than 7 kb (see the list in supporting online material for references and characteristics). These sequences were then merged with ‘classical’ *Hox* transcripts, grouped according to cluster and orientation and TUs were constructed using the Contig Assembly Program (http://www.infobiogen.fr/services/analyseq/cgi-bin/cap_in.pl) [26]. CAP generated contigs were then checked for misalignments. To identify putative homologuous TUs, non-redundant representative sequences for each TU were selected on the basis of the CAP contigs and blasted against vertebrate transcription databases.

In the case of the 14 antisense TUs, we collected a representative set of 52 sequences to identify putative homologous and to evaluate the coding potential. Using the Diogenes ORF prediction program (http://web.ahc.umn.edu/cgi-bin/diogenes/diogenes.cgi), eight different sequences presented a score compatible with an ORF (p>10-3) but subsequent BLAST analysis failed to detect any conserved pattern outside human.

These 52 sequences were systematically blasted against human database and alignment with sense *Hox* transcripts were reported as an indication of putative SAS contacts. Imperfect alignment and inconsistency in the genomic locations were the signs of putative *trans*-SAS contacts. Conservation of the SAS sequences was assessed by species cross-blasting. We undertook a similar procedure for the mouse *Hox* antisense TUs. The results are summarized in table 2.
